# Delivery of the selenoprotein thioredoxin reductase 1 to mammalian cells

**DOI:** 10.3389/fmolb.2022.1031756

**Published:** 2022-10-11

**Authors:** David E. Wright, Tarana Siddika, Ilka U. Heinemann, Patrick O’Donoghue

**Affiliations:** Departments of Biochemistry and Chemistry, The University of Western Ontario, London, ON, Canada

**Keywords:** cell biology, cell penetrating peptide, enzymology, genetic code expansion, redox biology, selenocysteine, thioredoxin reductase, trans-activator of transcription peptide

## Abstract

Over-expression of genetically encoded thioredoxin reductase 1 (TrxR1) TrxR1 can be toxic to cells due to the formation of a truncated version of the enzyme. We developed a new mammalian cell-based model to investigate TrxR1 activity. Fusion of the HIV-derived cell penetrating peptide (TAT) enabled efficient cellular uptake of purified TrxR1 containing 21 genetically encoded amino acids, including selenocysteine. The TAT peptide did not significantly alter the catalytic activity of TrxR1 *in vitro*. We monitored TrxR1-dependent redox activity in human cells using a TrxR1-specific red fluorescent live-cell reporter. Using programmed selenocysteine incorporation in *Escherichia coli*, our approach allowed efficient production of active recombinant human selenoprotein TrxR1 for delivery to the homologous context of the mammalian cell. The delivered TAT-TrxR1 showed robust activity in live cells and provided a novel platform to study TrxR1 biology in human cells.

## Introduction

Mammalian thioredoxin reductases (TrxR) are an important enzyme family that helps to protect cells from damage caused by reactive oxygen species (ROS) (Mustacich and Powis, 2000) and serves critical roles in redox signaling (Ren et al., 2017). TrxR1 is the most abundant member of the TrxR family and is expressed in the cytosol (Mustacich and Powis, 2000). TrxR1 is also a validated cancer target, and several efforts are underway to generate new inhibitors of TrxR1 that can compromise the rapid growth of cancer cells (Arnér, 2017; Hasan et al., 2022). For example, recent work on compounds with a 4,5-dichloropyridazinone core structure were identified as irreversible TrxR1 inhibitors that displayed potent toxicity in a series of cancer cells (Busker et al., 2020).

TrxR1 is a selenoprotein (Tamura and Stadtman, 1996), and its activity relies on the efficient incorporation of selenocysteine (Sec, U) in response to the UGA codon at position 550. TrxR1 is a complex enzyme that catalyzes the transfer of electrons from a bound NADPH cofactor, with the aid of a second FAD cofactor, to a disulfide N-terminal active site. From there, TrxR1 transfers electrons to the C-terminal selenylsulfide active site (GCUG). The enzyme then reduces the selenylsulfide to selenolthiol that subsequently acts to reduce oxidized substrates in the cell. Thus, the Sec residue forms a critical part of the TrxR1 active site and is necessary for its enzymatic reduction of the oxidized thioredoxin (Trx), a major TrxR1 substrate (Wright et al., 2018). The reduced Trx1 is then able to resolve oxidative damage in cellular proteins (Lee et al., 2013). TrxR1 itself is also able to directly reduce protein disulfide substrates (Lundstrom-Ljung et al., 1995) and small molecule ROS, including hydrogen peroxide (Zhong and Holmgren, 2000), lipoic acid (May et al., 2007), selenite (Kumar et al., 1992), and quinones (Xia et al., 2003).

Because of the vital role of TrxR1 in human biology and applications in medicine, there is a continuing need to generate mammalian cells with precise levels of TrxR1 activity. Applications of these cell lines will provide insight into how TrxR1 activity regulates redox biology in human cells and provide a critical platform to screen new inhibitors of TrxR1. One roadblock in this area involves the challenges of generating mammalian cell lines that over-express TrxR1. Unfortunately, normal approaches to genetically over-express TrxR1 in mammalian cells lead to increased cell death. Over-expression of TrxR1 by stable transfection produced a > 2-fold increase in cell death in MCF-7 cells compared to empty vector control (Ma et al., 2001). A form of TrxR1 that stops at the UGA550 codon, and lacks the C-terminal Sec-Gly dipeptide, is toxic and induces cell death in human A549 cells (Anestal and Arnér, 2003). In HEK 293T cells, one recent report generated a stable cell line over-expressing TrxR1, yet no experiments were provided to demonstrate incorporation of Sec in TrxR1 or to evaluate apoptosis (Lu et al., 2022). Direct lipid-mediated transfection of TrxR1 protein lacking the two C-terminal residues caused a significant increase in cell death, while transfection of the full-length and Sec-containing TrxR1 showed no change in cell toxicity (Anestal and Arnér, 2003). These observations may result from selenium compromised thioredoxin reductase-derived apoptotic proteins (SecTRAPS) (Anestal et al., 2008). Indeed, inhibition of genetically encoded TrxR1 in mammalian cells can also produce SecTRAPS and induce apoptosis (Zhang et al., 2022).

**FIGURE 1:**
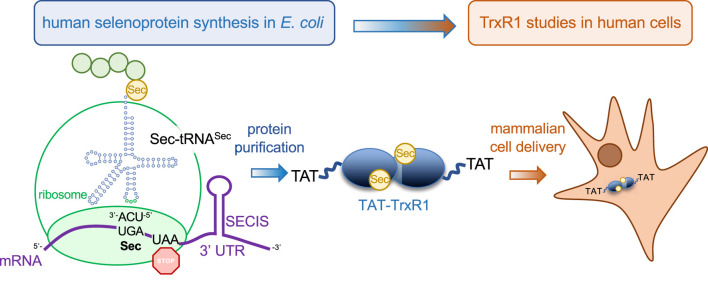
Production of recombinant selenocysteine containing TrxR1 in *E. coli* and delivery to mammalian cells. Recombinant production of human TrxR1 containing Sec550 was accomplished by fusing a bacterial Sec insertion sequence (SECIS) hairpin to the 3′ untranslated region of the human TrxR1 gene (Bröcker et al., 2014). We have previously confirmed that the approach leads to the stoichiometric installation of Sec and the production of abundant and active human TrxR1 from bacterial cells (Bröcker et al., 2014; Wright et al., 2018; Wright et al., 2021). The construct used here also contains an N-terminal TAT peptide fusion (YGRKKRRQRRRPQ) that enables efficient delivery of purified recombinant TAT-TrxR1 to mammalian cells.

We previously developed a genetic code expansion approach with Sec to generate recombinant human TrxR1 from *Escherichia coli* containing stoichiometric incorporation of Sec at position 550 (Figure 1) (Bröcker et al., 2014; Wright et al., 2018; Wright et al., 2021). Here we used the same approach and produced a novel form of recombinant human TrxR1 that also contains an N-terminal cell penetrating peptide (CPP) tag derived from the human immunodeficiency virus (HIV) Trans-Activator of Transcription (TAT) protein (Han et al., 2000; Vives et al., 2003; Lichtenstein et al., 2021). CPPs, such as the TAT peptide, include a diverse and growing catalog of small peptide tags that can be used to deliver proteins (Kurrikoff et al., 2021), mRNAs (Yokoo et al., 2021), and small molecules (Tian and Zhou, 2021), including pharmaceutical compounds, directly to mammalian cells (Shoari et al., 2021). We used a CPP tag to circumvent the need to genetically over-express TrxR1 due to the pitfalls associated with expressing TrxR1 in mammalian cells. Using a genetically encoded red fluorescent TrxR1 activity reporter (TrxRFP1) (Fan et al., 2017), we found that TAT-dependent cellular uptake was rapid and led to robust TrxR1 activity in mammalian cells. The TAT-tag provides a greatly needed platform to study TrxR1 biology and our technology will enable future studies to identify novel TrxR1 inhibitors for applications in cancer and other human diseases.

## Materials and methods

### Plasmids and molecular cloning

Wild-type TrxR1 was expressed in *E. coli* from the plasmid pET-TrxR1, previously described (Wright et al., 2018), that contains N-terminally His_6_-tagged human TrxR1 (isoform 4) with a UGA codon at residue Sec550. The TrxR1 gene is followed by selenocysteine insertion sequence (SECIS) RNA hairpin loop, which is derived from the *E. coli fdhF* gene, in the 3′ untranslated region (3′ UTR) (Bröcker et al., 2014; Wright et al., 2018; Wright et al., 2021). Site-specific insertion of Sec at the UGA550 codon depends on recruitment of the native *E. coli* Sec insertion machinery that decodes the UGA550 codon as Sec due to the adjacent SECIS in the 3’ UTR, as previously (Bröcker et al., 2014; Wright et al., 2018; Wright et al., 2021).

The bacterial expression vector pTAT-HA, a kind gift from Steven Dowdy (Addgene plasmid #35612), was used to produce TAT fused TrxR1 protein. The pTAT-HA vector includes an N-terminal His_6_-tag upstream of the adjacent TAT peptide sequence (YGRKKRRQRRR) (Nagahara et al., 1998). The TrxR1 gene including the 3′UTR SECIS was polymerase chain reaction (PCR) amplified using primers with *Nco*I and *Eco*RI restriction sites on the 5′ and 3′ ends, respectively. Following digestion of the pTAT-HA vector and TrxR1 insert, T4 DNA ligase (New England Biolabs, Ipswich, MA, USA) was used to ligate TrxR1 into the *Nco*I and *Eco*RI sites in the similarly digested pTAT-HA vector. The resulting construct was verified by DNA sequencing (Azenta Life Sciences, New Jersey, USA). Sequences of the TrxR1 and TAT-TrxR1 gene constructs are available in the Supplementary Data.

### TrxR1 protein purification

*E. coli* strain BL21 (DE3) was transformed with pET-TrxR1 or pTAT-TrxR1 plasmid. Following selection on lysogeny broth (LB) agar plates containing 100 μg/mL ampicillin, at least 3 independent single colonies were inoculated into separate overnight 10 mL pre-cultures in LB with 100 μg/mL ampicillin. The pre-cultures were then used to inoculate preparative 1 L cultures in LB containing 100 μg/mL ampicillin and 10 µM sodium selenite and were incubated with shaking at 37°C. We followed a previously optimized protocol for the production of selenoproteins in *E. coli* (Bröcker et al., 2014; Wright et al., 2018; Wright et al., 2021). At A_600_ = 1.2, the temperature was decreased to 20°C. At A_600_ = 1.5, 1 mM isopropyl β-d-1-thiogalactopyranoside (IPTG) was added to the culture with shaking for 18 h to induce protein production. Cell pellets were harvested by centrifugation and stored at -80°C until purification. TrxR1 proteins were purified as previously described (Wright et al., 2018). Briefly, cell pellets were resuspended in 30 ml phosphate buffer (100 mM potassium phosphate, pH 7.2, 10% glycerol), supplemented with lysozyme (1 mg/mL), and disrupted by sonication with 70% amplification. After centrifugation at 6,250 × *g* for 1 h, clear cell lysates were purified by affinity chromatography using Ni^2+^-Nitrilotriacetic acid (NTA) resin (HisPur Ni-NTA Resin, PI88222, ThermoFisher Scientific, Mississauga, ON, Canada) as detailed before (Wright et al., 2018). Purified TrxR1 proteins were kept in storage buffer containing 100 mM potassium phosphate pH 7.2 and 50% glycerol at -80°C until use.

### TrxR1 *in vitro* activity assays

TrxR1 activity was monitored, as we did before (Wright et al., 2018), using 5,5-dithio-bis-(2-nitrobenzoic acid) (DTNB) to detect the level of reductive activity from TrxR1 and TAT-TrxR1. Active TrxR1 reduces DTNB to 2-nitro-5-thiobenzoate (TNB^2-^), which absorbs at wavelength 414 nm (A_414_). Each reaction mixture contained 50 nM or 100 nM TrxR1, 300 µM Nicotinamide adenine dinucleotide phosphate (NADPH) and 5 mM DTNB in buffer containing 100 mM potassium phosphate, 1 mM Ethylenediaminetetraacetic acid (EDTA) pH 7.0. Protein concentration was assessed by the Bradford protein assay (Catalog #5000006; Bio-Rad) at 595 nm according to the manufacturer’s instructions. Reactions were initiated in a 96 well micro-plate by the addition of DTNB to the reaction mixture containing TrxR1 (at 50 nM or 100 nM as indicated) and NADPH in a final volume of 100 µL. Measurements were taken in a Biotek Synergy H1 microplate reader every 1 min for an 80 min time course. All assays were performed in triplicate using three independent enzyme reactions for each condition tested.

### Endogenous TrxR1 activity in live cells and fluorescence microscopy

A TrxR1 activity biosensor (TrxRFP1) was used to monitor the TrxR1 activity in live cells by measuring the changes in red fluorescence of the reporter (Fan et al., 2017). The pcDNA3-TrxRFP1 was a kind gift from Huiwang Ai (Addgene plasmid # 98996). HEK 293T cells were cultured in Dulbecco’s Modified Eagle Medium (Cellgro DMEM, ThermoFisher Scientific) containing 10% fetal bovine serum and 1% penicillin/streptomycin in 5% CO_2_ at 37°C. Equal numbers of HEK 293T cells were seeded onto a 6 well culture plates containing media and transfected with Lipofectamine 2000 (Invitrogen) and 2 μg/mL plasmid DNA bearing the TrxRFP1 reporter according to manufacturer’s instructions. In three biological and three technical replicates, un-transfected cells and transfected cells were monitored by fluorescent microscopy (excitation 542 ± 20 nm, emission 593 ± 40 nm) using an EVOS FL Cell Imaging System (ThermoFisher Scientific). At 24 h after transfection, cell images were acquired every 1 min for a 60 min time course. At the 10 min time point, TrxR1 inhibitor aurothioglucose (ATG) was added to the media at a concentration of 100 μM.

### TAT-TrxR1 delivery and activity in live cells

HEK 293T cells were transfected with the plasmid bearing the TrxRFP1 reporter as above. At 24 h after transfection, cells were incubated with no protein or with 0.5 μM of TrxR1 or TAT-TrxR1 protein. Immediately before addition of the protein and at 30 min and at 24 h after incubation with protein or no protein, bright field and fluorescent (excitation 542 ± 20 nm, emission 593 ± 40 nm) cell images for each condition were acquired EVOS FL Cell Imaging System. Fluorescence intensities were analyzed by image J software and statistical analysis was based on 3 biological replicates with 3 technical replicates each using pairwise single factor analysis of variance (ANOVA).

### Western blotting

HEK 293T cells were cultured in DMEM (Cellgro) media containing 10% fetal bovine serum and 1% penicillin/streptomycin in 5% CO_2_ at 37°C. Equal numbers of cells were plated onto 6 well culture plates and incubated with buffer only or with 1 μM TrxR1 or TAT-TrxR1 protein. After a 1-h incubation, cells were washed with phosphate buffered saline (PBS) and cell pellets were collected by centrifugation. Cells were resuspended in lysis buffer containing (50 mM Na_2_HPO_4_, 1 mM Na_4_P_2_O_7_, 20 mM NaF, 2 mM EDTA, 2 mM EGTA, 1 mM dithiothreitol, 300 µM phenylmethylsulfonyl fluoride (PMSF), and protease inhibitor tablet (Catalog number 04 693 159 001, Roche Canada, Mississauga, ON, Canada). Pellets were kept on ice for 10 min and vortexed every 2 min. Supernatant was collected from cells representing three biological replicates by centrifugation at 15,000 × *g* for 15 min at 4°C.

Protein concentration was measured by Bradford assay and extracted proteins were diluted to 5 μg/μL, and 50 µg of protein was separated using 10% sodium dodecyl sulfate-poly acrylamide gel electrophoresis (SDS-PAGE). The protein was transferred to a polyvinylidene difluoride (PVDF) membrane using Turbo-Blot Turbo transfer system (Bio-Rad). The membrane was blocked in PBS with 0.1% (v/v) Tween 20 (PBST) and 5% (w/v) skim milk powder for 1 h at room temperature. The membrane was immunoblotted with specific anti-TrxR1 (B-2) mouse monoclonal primary antibody (sc-28321, Santa Cruz Biotechnology, Santa Cruz, CA, USA) overnight at 4°C followed by 3 × 10 min washes with agitation in PBST with 1% (w/v) skim milk powder. The membrane was then incubated in fluorescent secondary antibody (Goat-anti-mouse #AQ127, Sigma Aldrich, Oakville, Canada) for 1 h at room temperature with agitation and washed 3 × 10 min in PBST. The membrane was stored in PBS for further analysis and bands were visualized using a LiCor (LiCor Biosciences, Lincoln, Nebraska USA) fluorescence imager (693 nm emission, 700 nm excitation). A glyceraldehyde 3-phosphate dehydrogenase (GAPDH) antibody (#ab8245, abcam, Toronto, Canada) and secondary fluorescent antibody (Goat-anti-mouse #AQ127, Sigma Aldrich, Oakville, Canada) were used to establish loading controls.

In identical and independent experiments, additional western blots were performed by chemiluminescence imaging with the His_6_ and Vinculin antibodies. Briefly, the membrane was immunoblotted with specific mouse anti-His antibody (#H1029; Sigma, Oakville, Canada) overnight at 4°C followed by 3 × 10 min washes with agitation in PBST with 1% (w/v) skim milk powder. The membrane was then incubated in sheep anti-mouse immunoglobulin G horseradish peroxidase linked antibody (#GENA931, GE Healthcare, Oakville, Canada) for 1 h at room temperature with agitation and washed 3 × 10 min in PBST. The membrane was stored in PBS for further analysis and chemiluminescent signal detection was performed on a ChemiDoc MP system (BioRad). Mouse anti-vinculin antibody (#sc25336, Santa Cruz Biotechnology, Dallas, Texas, USA) and secondary antibody sheep anti-mouse immunoglobulin G horseradish peroxidase linked antibody (#GENA931 GE Healthcare, Oakville, Canada) were used to establish loading controls.

## Results

### Purification and activity of TrxR1 and TAT-TrxR1 variants

In previous work (Bröcker et al., 2014; Wright et al., 2018; Wright et al., 2021), we developed a facile approach to produce human TrxR1 in *E. coli*. The method involves recombinant expression of the human TrxR1 gene with a bacterial SECIS derived from the *E. coli fdhF* gene that was appended to the 3’ UTR (Figure 1). As we have demonstrated using mass spectrometry (MS) (Bröcker et al., 2014; Wright et al., 2018), elemental analysis with inductively coupled plasma mass spectrometry (ICP-MS) (Bröcker et al., 2014; Wright et al., 2018), and biochemical activity (Bröcker et al., 2014; Wright et al., 2018; Wright et al., 2021), our method leads to a yield of ∼2 mg/L *E. coli* culture of active TrxR1 with stoichiometrically incorporated Sec at the UGA550 codon (Bröcker et al., 2014; Wright et al., 2018; Wright et al., 2021). We previously characterized the incorporation of Sec in recombinant TrxR1 using tandem MS/MS analysis to demonstrate Sec incorporation, and we also conducted ICP-MS, which demonstrated quantitative incorporation at a level of 98.5% Sec in the recombinant TrxR1 protein (Bröcker et al., 2014). In independent work, we again provided tandem MS/MS analysis to demonstrate Sec incorporation in TrxR1, and we also performed matrix-assisted laser desorption/ionization (MALDI)-MS analysis to demonstrate Sec incorporation at UGA550 (Wright et al., 2018). Finally, we again employed ICP-MS to demonstrate quantitative incorporation of Sec in the recombinant TrxR1 protein (Wright et al., 2018).

**FIGURE 2:**
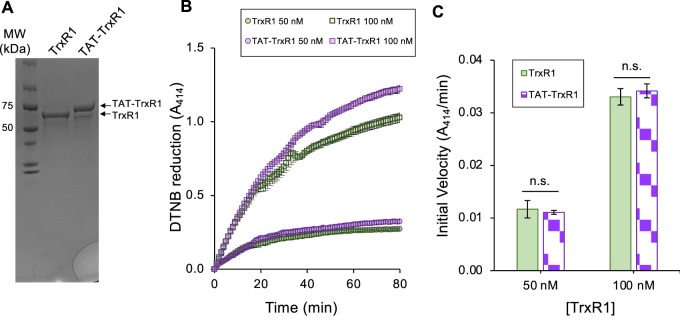
Purity and biochemical activity of TrxR1 and TAT-TrxR1 variants. The wild-type TrxR1 and TAT-tagged TrxR1 (TAT-TrxR1) were produced in *E. coli* according to our established approach (Figure 1) (Bröcker et al., 2014; Wright et al., 2018; Wright et al., 2021). Both the wild-type and TAT-TrxR1 variants were produced at high yield (∼2 mg/L *E coli* culture). **(A)** Coomassie stained gel shows the purity and molecular weights of TrxR1 and TAT-TrxR1. We used the reduction of Ellman’s reagent (DTNB) monitored by absorbance at 414 nm (A_414_) to assay **(B)** the activity of the wild-type (green curves) or TAT-TrxR1 (purple curves) at a concentration of 50 nM or 100 nM enzyme. Based on linear regression to the linear phase of the enzyme reactions, we derived **(C)** the initial velocity (A_414_/min) of each reaction and determined that there was no significant different between TrxR1 and TAT-TrxR1 activity at 50 nM or 100 nM concentrations. Error bars represent 1 standard deviation of 3 independent enzyme reactions and statistical analysis was based on pairwise single factor ANOVA (ns, not significant).

Here, we modified this original TrxR1 construct to include a TAT peptide at the N-terminus of the TrxR1 construct that produces a TAT-TrxR1 protein. Following purification, we confirm similar purity and yield of the TAT-TrxR1 protein compared to the wild-type TrxR1 enzyme (Figure 2A). We previously characterized recombinant TrxR1 activity using Ellman’s reagent (5,5′-Disulfanediylbis (2-nitrobenzoic acid, DTNB), 9,10-phenanthrequinone, as well as an insulin linked Trx1 assay (Wright et al., 2018). We found that recombinant TrxR1 showed similar and robust activity with each of these substrates (Wright et al., 2018). Because of this fact and because reduction of DTNB is a straightforward assay for TrxR1 activity that is monitored at A_414_ by the yellow color of the reduced form of DTNB (2-nitro-5-thiobenzoate anion, TNB^2-^), we chose to characterize the TAT-TrxR1 activity using DTNB.

Thus, to compare the activity of the wild-type and TAT-tagged TrxR1 enzymes, we measured the DTNB reduction activity of TrxR1 and TAT-TrxR1 at 50 nM and 100 nM concentrations over a time course (Figure 2B). In comparing the initial velocity of the reactions at 50 nM or 100 nM TrxR1 or TAT-TrxR1 concentrations, we recorded no significant difference between the wild-type or TAT-tagged TrxR1 proteins (Figure 2C). The TAT-TrxR1 has activity that is robust and indistinguishable from the recombinant TrxR1 protein and because the activity of TrxR1 relies critically on Sec550 incorporation, we can confirm the TAT-TrxR1 protein contains the same level of Sec as in TrxR1, which we documented before using multiple mass spectrometry methods (Bröcker et al., 2014; Wright et al., 2018). Thus, the TAT-tag does not significantly affect that activity of the TrxR1 enzyme, and the TAT-tagged version of the protein will provide an appropriate model of TrxR1 activity that can be readily delivered to mammalian cells.

### Assessing the TrxRFP1 reporter in mammalian cells

**FIGURE 3:**
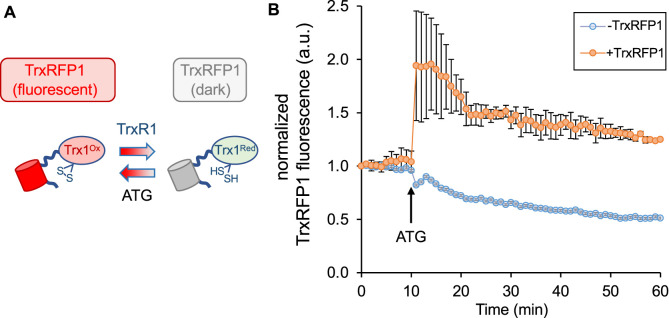
Measurement of endogenous TrxR1 activity in live cells. HEK 293T cells were transfected with a plasmid expressing a TrxR1 specific activity reporter. **(A)** The reporter (TrxRFP1) is a fusion of RFP with the TrxR1 substrate protein Trx1 (Fan et al., 2017). In the oxidized state (Trx1^Ox^) the fused RFP (red cylinder) is fluorescent, while in the reduced state (Trx1^Red^) the RFP fluorescence (gray cylinder) is lost in proportion to the amount of TrxR1 activity. **(B)** The time course begins 24 h after transfection and records TrxRFP1 fluorescence (excitation 542 ± 20 nm, emission 593 ± 40 nm) in HEK 293T cells transfected with (orange circles) or without (blue circles) the plasmid encoding TrxRFP1. Following application of TrxR1 inhibitor aurothioglucose (ATG) at 10 min after we began recording red fluorescence, cells with the TrxRFP1 reporter show a strong induction of red fluorescence that is proportional to the level of TrxR1 inhibition. Error bars represent ±1 standard deviation of three biological replicates.

Before attempting to deliver the TrxR1 or TAT-TrxR1 proteins to mammalian cells, we characterized the response of a live-cell reporter for TrxR1 activity. The TrxRFP1 reporter was engineered and developed previously (Fan et al., 2017). We obtained a plasmid that genetically encodes the TrxRFP1 reporter, which is a fusion protein of RFP with the natural substrate of TrxR1, Trx. In TrxRFP1, Trx1 was fused to the N-terminal of an engineered RFP protein where native cysteine residues (Cys) were replaced in the protein and a new redox coupled pair of Cys residues was introduced near the N- and C-terminus of the RFP domain. Thus, following reduction of the Trx1 domain by TrxR1, the Trx1 moiety can then reduce the engineered Cys redox couple in the RFP domain and eliminate red fluorescence of RFP. Once expressed in mammalian cells, TrxRFP1 fluorescence is inversely correlated with TrxR1 activity. In the reduced form, the Trx1 domain of the reporter disrupts the normal red fluorescence of the fused RFP, while in the oxidized form of Trx1 the red fluoresce of RFP is restored in proportion to the amount of oxidized Trx1. Because Trx1 is a selective substrate of TrxR1 (Fan et al., 2017), the system provides a specific and robust biosensor to monitor TrxR1 activity in live cells (Figure 3A).

To test the reporter in our hands, we transiently transfected the plasmid bearing TrxRFP1 into HEK 293T cells, and we used cells transfected without plasmid as a control. At 24 h after transfection, we began monitoring red fluorescence in the control cells and in cells transfected with the TrxRFP1 plasmid. After 10 min, we then added the well-established TrxR1 inhibitor aurothioglucose (ATG) (Gromer et al., 1998) to the media at a concentration of 100 μM. Using fluorescence microscopy, we observed a sharp increase in red fluorescence across 3 biological replicates, only in cells expressing the TrxRFP1 reporter (Figure 3B). The data demonstrate inhibition of endogenous TrxR1 in live HEK 293T cells, and in agreement with previous observations (Fan et al., 2017), confirm the proper function of the TrxRFP1 reporter.

### Delivery of TAT-TrxR1 to mammalian cells

As noted above, genetic approaches to over-express TrxR1 in mammalian cells can lead to substantial cytotoxicity, and lipid-based protein transfection approaches with TrxR1 (Anestal and Arnér, 2003) or other proteins (Suhorutsenko et al., 2011) are of limited efficiency. Furthermore, lipid-based transfection reagents also induce toxicity and immunogenic reactions in mammalian cells (Suhorutsenko et al., 2011), demonstrating the need for an improved approach to deliver active TrxR1 to cells.

**FIGURE 4:**
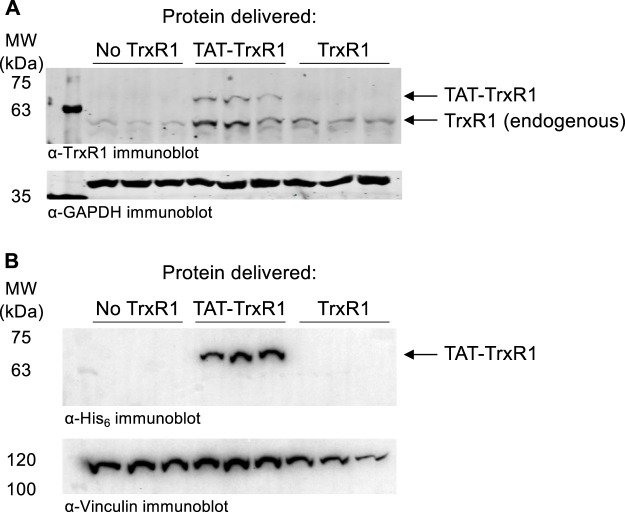
Immunoblotting confirms delivery of TAT-TrxR1 to cells. **(A)** Biological triplicates of HEK 293T cells were incubated for 1 h with buffer only (No TrxR1), with TAT-tagged TrxR1 (TAT-TrxR1), or with TrxR1 lacking the TAT-tag (TrxR1). Following incubation, cells werewashed to remove any protein remaining outside of the cells. The location of endogenous TrxR1 and TAT-tagged TrxR1 are indicated in a western blot with anti-TrxR1 antibody (above), and GAPDH was used as a loading control (below). **(B)** In an independent set of biological triplicates an identical experiment was performed. HEK 293T cell were incubated for 1 h with buffer only (No TrxR1), with TAT-tagged TrxR1 (TAT-TrxR1), or with TrxR1 lacking the TAT-tag (TrxR1). Following washing of the cells, a western blot was performed with the anti-His_6_ antibody as well as the anti-Vinculin antibody as a loading control. Both TAT-TrxR1 and TrxR1 protein contain the His_6_ tag, yet only the TAT-TrxR1 protein was imported into the cells.

In the absence of any transfection reagents, we incubated HEK 293T cells with buffer only, with wild-type TrxR1, or with TAT-tagged TrxR1. At 1 h after incubation with the proteins, cells were washed to remove any extracellular protein and analyzed by western blotting with anti-TrxR1 and with anti-GAPDH as a loading control (Figure 4A). In the case of TAT-TrxR1, we observed a consistent level of successfully delivered TAT-TrxR1 to cells, as evident by the larger molecular weight of TAT-TrxR1 compared to the endogenous protein.

To confirm selective uptake of the TAT-TrxR1, we also performed western blotting of an identical and independent set of biological triplicate experiments using the anti-His_6_ antibody and anti-Vinculin antibody as a loading control. As above, at 1 h after incubation of HEK 293T cells with buffer only, with wild-type TrxR1, or with TAT-tagged TrxR1, cells were washed to remove any protein remaining outside of the cells. Both the TAT-TrxR1 and TrxR1 recombinant proteins contain the His_6_ tag. We detected only the TAT-TrxR1 as successfully delivered to the interior of the cells (Figure 4B).

### TAT-tagged dependent uptake and activity of TAT-TrxR1 in live cells

**FIGURE 5:**
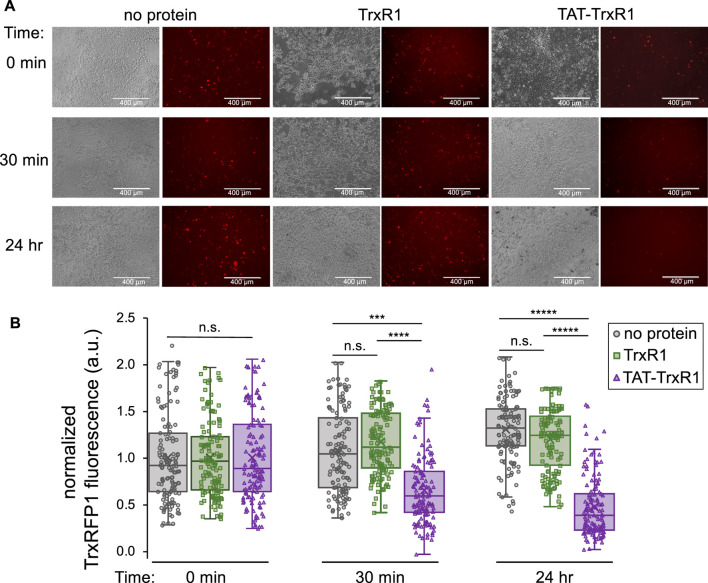
TAT-dependent delivery of active TrxR1 to mammalian cells. At 24 h after transfection with a plasmid bearing the TrxRFP1 activity reporter, cells were incubated with buffer only (no protein), with wild-type TrxR1 lacking the TAT-tag (TrxR1), or with TAT-tagged TrxR1 (TAT-TrxR1). **(A)** Following addition of protein to the media, brightfield images of the cells and fluorescence images of the TrxRFP1 reporter (excitation 542 ± 20 nm, emission 593 ± 40 nm) were recorded before addition of protein (0 min), at 30 min, and at 24 h after protein delivery. Decreasing red fluorescence demonstrates increased TrxR1 activity inside the cells. **(B)** At the 0 min time point, the average value of the fluorescence was normalized to 1.0 across each condition. Only the cells incubated with TAT-TrxR1 displayed a time dependent and statistically significant decrease in TrxRFP1 fluorescence during the time course. Data points taken from individual cells treated with no protein (gray circles), TrxR1 (green squares), or TAT-TrxR1 (purple triangles) are shown overlaid with box and whisker plots indicating the mean (x), median (line), range (bars), and lower and upper quartiles (boxed) of the data. Statistical analysis was based on 3 biological replicates and 3 technical replicates each and pairwise single factor ANOVA (ns, not significant; ***p < 0.005; ****p < 0.0001; *****p < 10^–5^).

To determine if the successfully delivered TAT-TrxR1 was active in HEK 293T cells, we conducted a series of experiments using the TrxRFP1 reporter examined above. First, HEK 293T cells were transfected with the plasmid encoding the TrxRFP1 reporter. At 24 h after transfection, cells were incubated with buffer only, with wild-type TrxR1, or with TAT-TrxR1. We then monitored the changes in red fluorescence of the TrxRFP1 reporter at 0 min, 30 min, and 24 h after incubation with the TrxR1 protein variants or with no protein added. At each time point, both brightfield and fluorescent images of the cells were captured and quantitated (Figure 5). As anticipated, in the case of the wild-type TrxR1 and the no protein control, we found no significant change in the TrxRFP1 fluorescence over the 24-h time course. In contrast, only the cells incubated with TAT-TrxR1 showed significant and marked decreases in red fluorescence (Figure 5).

These data agree precisely with our findings based on western blotting (Figure 4). Because only the TAT-TrxR1 was delivered successfully to the interior of the cells, only cells treated with TAT-TrxR1 and not those treated with TrxR1 showed a significant increase in TrxR1-specific activity. Thus, together the data demonstrate that only incubation with TAT-TrxR1 led to robust TrxR1-specific reduction activity inside the live human cells. The TAT-tag, therefore, represents a facile strategy to deliver active human TrxR1 selenoprotein to mammalian cells.

As a first demonstration of the TAT-tagging approach to delivery selenoprotein to human cells, we chose to work with HEK 293T cells. These cells are well established cell biological model systems that display robust growth and high transfection efficiencies (Thomas and Smart, 2005). This aspect was important since we relied on the TrxRFP1 live cell reporter to demonstrate the activity from the TAT-TrxR1 protein delivered to the human cells. Furthermore, since HEK 293T cells are derived from kidney cells, where endogenous TrxR1 is abundant and active (see Figures 3, 4), these particular cells are also appropriate models systems to study TrxR1 biology (Zhu et al., 2022), including in the context of chemotherapeutics where TrxR1 has been implicated as a major source of adverse effects of cisplatin compounds (Cheng et al., 2020). In the future, we will apply these approaches to other human and mammalian cell lines.

## Discussion

### Genetic code expansion and codon reassignment

Genetic code expansion is a cornerstone application in synthetic biology that enables protein production with amino acid building blocks beyond the canonical 20 amino acids that are normally used to make proteins (O'Donoghue et al., 2013). Here we used a genetic code expansion approach to incorporate the 21st amino acid, Sec, into a recombinant human protein produced in *E. coli*. The genetic code expansion method, which we established previously (Bröcker et al., 2014; Wright et al., 2018; Wright et al., 2021), involves expression of a synthetic gene construct where we fused SECIS from the 3′UTR of an *E. coli fdhF* gene to a human TrxR1 gene that we codon optimized for *E. coli*. The resulting protein product is an active TrxR1 enzyme containing 21 different amino acids. As a novel extension of our approach, here we employed the CPP TAT-tag to enable our studies of the recombinant human TrxR1 enzyme in the homologous context of human cells without any engineering steps to otherwise modify the human cells themselves.

Genetic code expansion systems enable site-specific incorporation of non-canonical amino acids (ncAAs) into proteins to introduce 21 (Bain et al., 1992; Bröcker et al., 2014; Haruna et al., 2014), 22 (Köhrer et al., 2003; Venkat et al., 2018; Wright et al., 2018; Wright et al., 2021), or recently, in a few cases, 23 different amino acids into proteins (Dunkelmann et al., 2020; Tharp et al., 2021). These approaches, which normally require engineering a new tRNA synthetase and tRNA pair, are powerful in their ability to introduce new chemistry into proteins and functionalities that enable bio-orthogonal protein labeling (Ngo and Tirrell, 2011). Applications include creating new probes for protein function (O'Donoghue et al., 2021; Courtney and Deiters, 2018) and protein structure (Mihaila et al., 2022) as well as protein and peptide pharmaceuticals to provide novel antibiotics (Vinogradov and Suga, 2020), vaccines (Si et al., 2016), and to treat major human diseases, including cancers (Huang and Liu, 2018) and auto-immune disorders (Gauba et al., 2011).

Despite the success of genetic code expansion and its many applications in biology as well as studies of health and disease, the methods often involve re-assignment of a particular codon, normally one or more of the stop codons (UGA, UAA, and UAG), not only in a particular protein of interest but also in every instance of that codon throughout the proteome. Indeed, this situation leads to the extension of many natural proteins beyond their normal termination point (Heinemann et al., 2012). When used in a cell free context (Ranji Charna et al., 2022), or simply in a production host to make proteins for downstream applications, this defect is of less importance. Genetic code expansion approaches, however, were also deployed in the very same cells that are the exact object of study. In one example, expanding the genetic code by reassigning the UAG codon to *N*_*ε*_-acetyl-lysine in mammalian cells to study the impact of a specific histone modification also caused transcriptome-wide changes in gene expression ranging from 2 to 100-fold (Elsasser et al., 2016). Thus, the engineered cells were substantially more affected by the codon reassignment than by the insertion of a specific acetylation into histone proteins. The combination of CPPs with genetic code expansion that we demonstrated here allowed engineering in one kind of cell and then subsequent delivery of a protein product of genetic code expansion into otherwise naïve mammalian cells, thus minimizing perturbations associated with expanding the genetic code.

### Delivery of ncAA-containing proteins with cell penetrating peptides

Since genetic code expansion can have such a dramatic impact on the nature of the cell under investigation, we sought to devise a novel approach to deliver proteins containing ncAAs to mammalian cells, eliminating the need to engineer the mammalian cells themselves. CPPs represent a diverse and growing catalog of small peptides that have already demonstrated great utility in facilitating the delivery of proteins (Kurrikoff et al., 2021), mRNAs (Yokoo et al., 2021), and small molecules (Tian and Zhou, 2021) to mammalian cells (Shoari et al., 2021). The TAT peptide is derived from the HIV TAT protein, which is essential for HIV replication (Arya et al., 1985). A chemically synthetized 86 amino acid full-length TAT protein was rapidly taken up by HeLa cells, and the protein delivery activity was localized to the region including residues 37-72 of the synthetic TAT protein (Green and Loewenstein, 1988). Shortly thereafter, this same region of the TAT protein was chemically cross-linked to several different larger proteins, including beta-galactosidase, horseradish peroxidase, RNase A, and domain III of *Pseudomonas* exotoxin A that were all shown to be rapidly delivered to several different mammalian cell types in culture (Fawell et al., 1994).

Our work demonstrated a novel approach to deliver an active and full-length human selenoprotein to mammalian cells *via* fusion with a genetically encoded CPP for the first time. We found that the selenoprotein TAT-TrxR1 was efficiently delivered to cells and already displayed significant activity 30 min after incubation of the protein with HEK 293T cells. We found the TAT-TrxR1 continued to display robust and TrxR1-specific reduction activity in the cells for at least 24 h after protein delivery. To our knowledge, there are no other examples in the literature using cell penetrating peptides to deliver Sec-containing full-length proteins to mammalian cells. A recent study successfully engineered a Sec-containing small peptide, called PSELT (FQICVSUGYR), and demonstrated efficient delivery of the peptide to human neuroblastoma SH-SY5Y cells (Alsharif et al., 2021). PSELT protected the cells against oxidative damage associated with Parkinson’s disease, indicating an important avenue for therapeutic application of Sec-containing peptides.

Indeed, there is just one example of delivering ncAA-containing protein to mammalian cells using a CPP tag. The report demonstrated production of the murine dihydrofolate reductase (DHFR) in *E. coli* containing the ncAA *N*_ε_-propargyloxycarbonyl-lysine (Bi et al., 2018). The authors then used click chemistry along with an azide-functionalized TAT peptide to chemically link the TAT-peptide to the DHFR protein, achieving a 90% yield. The DHFR protein was also chemically cyclized to help prevent degradation in HeLa cells. Although the engineered DHFR protein showed relatively efficient delivery to the cells at 1 h after incubation with the protein, by the 2-h time point most of the protein was degraded. In the case of the linear or non-cyclized TAT functionalized DHFR, nearly all of the protein was degraded after 2 h (Bi et al., 2018). Thus, our work demonstrates additional novelty in that the TAT-tag was genetically encoded in *E. coli* in the same protein as the ncAA Sec, and no downstream chemical synthetic steps were required. Our simpler approach also showed a longer lasting effect as we observed robust activity of the TAT-TrxR1 protein up to the 24-h time point.

### A new system to study TrxR1-dependent activity in mammalian cells

Our approach to engineering selenoproteins production in *E. coli* has allowed us to develop a new system to study TrxR1 redox biology in a homologous context of the mammalian cell without engineering the mammalian cells. Our approach also requires no addition of costly and toxic transfection reagents. Previous work found that lipid-mediated transfection of TrxR1 was achieved but with limited efficiency (Anestal and Arnér, 2003). Indeed, compared to standard protein transfection reagents, including lipofectamine derivates, cell penetrating peptides, including TAT are well tolerated in mammalian cells. While lipofectamine can strongly depress cell viability, induced apoptosis, and generate significant immune responses in human acute monocytic leukemia cells (THP-1), cell penetrating peptides were found to be neither toxic nor immunogenic (Suhorutsenko et al., 2011).

## Conclusion

We demonstrated a simple and efficient approach to deliver the active human selenoprotein TrxR1 to mammalian cells. We showed that a genetically encoded TAT-tagged TrxR1 was produced in *E. coli* with similar efficiency and activity to an untagged TrxR1 in *E. coli* that we already demonstrated had stoichiometric incorporation of Sec550 by multiple methods (Bröcker et al., 2014; Wright et al., 2018; Wright et al., 2021). The TAT-tag enabled rapid delivery of the active selenoprotein to mammalian cells that provided robust Trx1-specific reductive activity for at least 24 h. Given the complex nature of selenoprotein synthesis in mammalian cells, our approach represents an important step forward to aid studies in the native and homologous context of mammalian cells of other human selenoproteins as well as ncAA-containing proteins produced in engineered cells with expanded genetic codes. Recent breakthroughs also enable site-specific incorporation of Sec at any location in a recombinant protein in *E. coli* (Haruna et al., 2014; Liu et al., 2018; Thyer et al., 2018; Welegedara et al., 2018; Wang et al., 2021), thus indicating that our approach with cell penetrating peptides can be applied to other selenoproteins and in combination with other genetic code expansion systems.

Finally, because TrxR1 is an important and validated drug target for cancers (Arnér, 2017; Busker et al., 2020; Hasan et al., 2022) and other human diseases, including Rheumatoid arthritis (Duan et al., 2022) and neurodegeneration (Wang et al., 2022), the system we developed here will not only be a valuable tool for studying TrxR1 biology in human cells, but also represents a novel platform to screen inhibitors of TrxR1. Since TrxR1 inhibitors have already found application in clinical settings (Sachweh et al., 2015; Zhang et al., 2021), our approach will facilitate applications in developing the next generation of therapeutics to target TrxR1 activity in human diseases.

## Data Availability

The original contributions presented in the study are included in the article/Supplementary Material, further inquiries can be directed to the corresponding author.
