# Supplementary material for: Delivery of the selenoprotein thioredoxin reductase 1 to mammalian cells
Source: Front Mol Biosci. 2022 Oct 11;9:1031756. doi: 10.3389/fmolb.2022.1031756 (PMC9595596; doi:10.3389/fmolb.2022.1031756)
Supplement: Supplementary file 1 [file DataSheet1.DOCX]

Supplementary Material for:

Delivery of the selenoprotein thioredoxin reductase 1 to mammalian cells

David E. Wright^1,†^, Tarana Siddika^1,†^, Ilka U. Heinemann^1^, & Patrick O’Donoghue^1,2,*^

^1^Departments of Biochemistry and ^2^Chemistry,

The University of Western Ontario, London, Ontario, Canada

†These authors contributed equally.

*Correspondence:
Patrick O’Donoghue
[patrick.odonoghue@uwo.ca](mailto:patrick.odonoghue@uwo.ca)

# Supplementary Data: DNA and protein sequences for TrxR1 constructs

**Human His6-TrxR1 (*E. coli* codon optimized) DNA sequence in pET-TrxR1 vector (1)**

ATGGGCAGCAGCCATCACCATCATCACCACAGCCAGGATCCGAATTCGTCCTGCGAAGACGGTCGTGCGCTGGAAGGCACCCTGTCCGAACTGGCTGCAGAAACGGACCTGCCGGTGGTATTTGTTAAACAGCGTAAAATCGGCGGCCACGGTCCGACTCTGAAAGCGTACCAGGAGGGTCGTCTGCAGAAACTGCTGAAAATGAACGGCCCGGAAGATCTGCCGAAATCTTACGATTACGATCTGATCATCATCGGCGGTGGTTCTGGTGGCCTGGCAGCCGCAAAGGAGGCAGCACAGTATGGCAAAAAAGTAATGGTACTGGACTTCGTTACCCCGACCCCACTGGGTACCCGTTGGGGCCTGGGTGGTACCTGCGTTAACGTAGGTTGCATTCCGAAAAAACTGATGCACCAGGCTGCCCTGCTGGGCCAGGCTCTGCAGGACTCCCGTAACTACGGTTGGAAAGTGGAAGAAACGGTTAAACATGATTGGGACCGCATGATCGAGGCGGTTCAAAACCACATCGGTAGCCTGAACTGGGGCTACCGCGTTGCACTGCGCGAGAAAAAAGTTGTATATGAAAACGCGTACGGTCAGTTCATTGGCCCGCACCGTATCAAAGCGACTAACAACAAAGGCAAGGAAAAAATTTACAGCGCAGAACGTTTCCTGATCGCGACCGGCGAACGTCCGCGTTACCTGGGTATCCCGGGCGATAAAGAATACTGTATCAGCTCCGATGACCTGTTTTCTCTGCCGTACTGCCCTGGCAAAACTCTGGTCGTGGGTGCATCCTACGTCGCTCTGGAGTGTGCAGGTTTCCTGGCAGGTATTGGCCTGGACGTTACCGTTATGGTGCGTTCTATTCTGCTGCGTGGTTTCGACCAAGATATGGCGAACAAAATCGGTGAACACATGGAAGAACACGGTATTAAATTCATCCGTCAGTTCGTTCCGATCAAAGTAGAACAGATTGAAGCCGGTACCCCGGGCCGTCTGCGTGTGGTAGCTCAGTCCACCAACAGCGAGGAAATTATCGAAGGCGAATACAACACTGTTATGCTGGCGATCGGTCGCGATGCTTGTACCCGTAAAATTGGCCTGGAAACGGTAGGCGTTAAAATCAATGAAAAAACCGGTAAAATTCCAGTAACCGACGAAGAACAGACCAACGTTCCGTATATCTATGCAATTGGTGATATCCTGGAGGATAAAGTTGAACTGACCCCTGTTGCTATCCAGGCTGGCCGCCTGCTGGCACAGCGTCTGTATGCCGGTAGCACCGTGAAGTGCGACTACGAAAACGTGCCGACCACGGTTTTCACCCCTCTGGAATATGGCGCATGCGGTCTGTCTGAAGAGAAAGCCGTAGAAAAATTCGGCGAAGAAAACATTGAAGTGTATCACTCTTATTTCTGGCCGCTGGAATGGACTATTCCGTCTCGTGACAACAACAAATGCTACGCTAAAATTATCTGCAACACGAAAGATAACGAACGTGTGGTTGGCTTCCACGTGCTGGGCCCGAATGCGGGTGAAGTGACTCAAGGTTTCGCGGCTGCCCTGAAATGCGGCCTGACGAAAAAGCAGCTGGATTCTACCATCGGTATCCATCCGGTGTGTGCTGAAGTTTTCACCACTCTGTCTGTCACCAAACGTAGCGGTGCGTCCATCCTGCAAGCAGGATGCTGAGGCTAATA**ATCGGTTGCAGGTCTGCACCAATCG**

TGA codon = Sec

TAA stop codon

*E. coli* fdhF **SecIS**

**Human His6-TrxR1 protein sequence**

MGSSHHHHHHSQDPNSSCEDGRALEGTLSELAAETDLPVVFVKQRKIGGHGPTLKAYQEGRLQKLLKMNGPEDLPKSYDYDLIIIGGGSGGLAAAKEAAQYGKKVMVLDFVTPTPLGTRWGLGGTCVNVGCIPKKLMHQAALLGQALQDSRNYGWKVEETVKHDWDRMIEAVQNHIGSLNWGYRVALREKKVVYENAYGQFIGPHRIKATNNKGKEKIYSAERFLIATGERPRYLGIPGDKEYCISSDDLFSLPYCPGKTLVVGASYVALECAGFLAGIGLDVTVMVRSILLRGFDQDMANKIGEHMEEHGIKFIRQFVPIKVEQIEAGTPGRLRVVAQSTNSEEIIEGEYNTVMLAIGRDACTRKIGLETVGVKINEKTGKIPVTDEEQTNVPYIYAIGDILEDKVELTPVAIQAGRLLAQRLYAGSTVKCDYENVPTTVFTPLEYGACGLSEEKAVEKFGEENIEVYHSYFWPLEWTIPSRDNNKCYAKIICNTKDNERVVGFHVLGPNAGEVTQGFAAALKCGLTKKQLDSTIGIHPVCAEVFTTLSVTKRSGASILQAGCUG

U = Sec residue

**Human His6-TAT-TrxR1 (*E. coli* codon optimized) DNA sequence in pTAT-HA vector** **(2)**

ATGCGGGGTTCTCATCATCATCATCATCATGGTATGGCTAGCATGACTGGTGGACAGCAAATGGGTCGGGATCTGTACGACGATGACGATAAGGATCGATGGGGATCCAAGCTTGGCTACGGCCGCAAGAAACGCCGCCAGCGCCGCCGCGGTGGATCCACCATGTCCGGCTATCCATATGACGTCCCAGACTATGCTGGCTCCATGGCCTCCTGCGAAGACGGTCGTGCGCTGGAAGGCACCCTGTCCGAACTGGCTGCAGAAACGGACCTGCCGGTGGTATTTGTTAAACAGCGTAAAATCGGCGGCCACGGTCCGACTCTGAAAGCGTACCAGGAGGGTCGTCTGCAGAAACTGCTGAAAATGAACGGCCCGGAAGATCTGCCGAAATCTTACGATTACGATCTGATCATCATCGGCGGTGGTTCTGGTGGCCTGGCAGCCGCAAAGGAGGCAGCACAGTATGGCAAAAAAGTAATGGTACTGGACTTCGTTACCCCGACCCCACTGGGTACCCGTTGGGGCCTGGGTGGTACCTGCGTTAACGTAGGTTGCATTCCGAAAAAACTGATGCACCAGGCTGCCCTGCTGGGCCAGGCTCTGCAGGACTCCCGTAACTACGGTTGGAAAGTGGAAGAAACGGTTAAACATGATTGGGACCGCATGATCGAGGCGGTTCAAAACCACATCGGTAGCCTGAACTGGGGCTACCGCGTTGCACTGCGCGAGAAAAAAGTTGTATATGAAAACGCGTACGGTCAGTTCATTGGCCCGCACCGTATCAAAGCGACTAACAACAAAGGCAAGGAAAAAATTTACAGCGCAGAACGTTTCCTGATCGCGACCGGCGAACGTCCGCGTTACCTGGGTATCCCGGGCGATAAAGAATACTGTATCAGCTCCGATGACCTGTTTTCTCTGCCGTACTGCCCTGGCAAAACTCTGGTCGTGGGTGCATCCTACGTCGCTCTGGAGTGTGCAGGTTTCCTGGCAGGTATTGGCCTGGACGTTACCGTTATGGTGCGTTCTATTCTGCTGCGTGGTTTCGACCAAGATATGGCGAACAAAATCGGTGAACACATGGAAGAACACGGTATTAAATTCATCCGTCAGTTCGTTCCGATCAAAGTAGAACAGATTGAAGCCGGTACCCCGGGCCGTCTGCGTGTGGTAGCTCAGTCCACCAACAGCGAGGAAATTATCGAAGGCGAATACAACACTGTTATGCTGGCGATCGGTCGCGATGCTTGTACCCGTAAAATTGGCCTGGAAACGGTAGGCGTTAAAATCAATGAAAAAACCGGTAAAATTCCAGTAACCGACGAAGAACAGACCAACGTTCCGTATATCTATGCAATTGGTGATATCCTGGAGGATAAAGTTGAACTGACCCCTGTTGCTATCCAGGCTGGCCGCCTGCTGGCACAGCGTCTGTATGCCGGTAGCACCGTGAAGTGCGACTACGAAAACGTGCCGACCACGGTTTTCACCCCTCTGGAATATGGCGCATGCGGTCTGTCTGAAGAGAAAGCCGTAGAAAAATTCGGCGAAGAAAACATTGAAGTGTATCACTCTTATTTCTGGCCGCTGGAATGGACTATTCCGTCTCGTGACAACAACAAATGCTACGCTAAAATTATCTGCAACACGAAAGATAACGAACGTGTGGTTGGCTTCCACGTGCTGGGCCCGAATGCGGGTGAAGTGACTCAAGGTTTCGCGGCTGCCCTGAAATGCGGCCTGACGAAAAAGCAGCTGGATTCTACCATCGGTATCCATCCGGTGTGTGCTGAAGTTTTCACCACTCTGTCTGTCACCAAACGTAGCGGTGCGTCCATCCTGCAAGCAGGATGCTGAGGCTAATA**ATCGGTTGCAGGTCTGCACCAATCG**

TAT DNA sequence

TGA codon = Sec

TAA stop codon

*E. coli* fdhF **SecIS**

**Human His6-TAT-TrxR1 protein sequence**

MRGSHHHHHHGMASMTGGQQMGRDLYDDDDKDRWGSKLGYGRKKRRQRRRGGSTMSGYPYDVPDYAGSMASCEDGRALEGTLSELAAETDLPVVFVKQRKIGGHGPTLKAYQEGRLQKLLKMNGPEDLPKSYDYDLIIIGGGSGGLAAAKEAAQYGKKVMVLDFVTPTPLGTRWGLGGTCVNVGCIPKKLMHQAALLGQALQDSRNYGWKVEETVKHDWDRMIEAVQNHIGSLNWGYRVALREKKVVYENAYGQFIGPHRIKATNNKGKEKIYSAERFLIATGERPRYLGIPGDKEYCISSDDLFSLPYCPGKTLVVGASYVALECAGFLAGIGLDVTVMVRSILLRGFDQDMANKIGEHMEEHGIKFIRQFVPIKVEQIEAGTPGRLRVVAQSTNSEEIIEGEYNTVMLAIGRDACTRKIGLETVGVKINEKTGKIPVTDEEQTNVPYIYAIGDILEDKVELTPVAIQAGRLLAQRLYAGSTVKCDYENVPTTVFTPLEYGACGLSEEKAVEKFGEENIEVYHSYFWPLEWTIPSRDNNKCYAKIICNTKDNERVVGFHVLGPNAGEVTQGFAAALKCGLTKKQLDSTIGIHPVCAEVFTTLSVTKRSGASILQAGCUG

U = Sec residue

TAT peptide

# Supplementary References

1. D. E. Wright, Z. Altaany, Y. Bi, Z. Alperstein and P. O'Donoghue: Acetylation Regulates Thioredoxin Reductase Oligomerization and Activity. *Antioxid Redox Signal*, 29(4), 377-388 (2018) doi:10.1089/ars.2017.7082

2. H. Nagahara, A. M. Vocero-Akbani, E. L. Snyder, A. Ho, D. G. Latham, N. A. Lissy, M. Becker-Hapak, S. A. Ezhevsky and S. F. Dowdy: Transduction of full-length TAT fusion proteins into mammalian cells: TAT-p27Kip1 induces cell migration. *Nat Med*, 4(12), 1449-52 (1998) doi:10.1038/4042
